# Association of Serum Adiponectin, Leptin, and Resistin Concentrations with the Severity of Liver Dysfunction and the Disease Complications in Alcoholic Liver Disease

**DOI:** 10.1155/2013/148526

**Published:** 2013-10-23

**Authors:** Beata Kasztelan-Szczerbinska, Agata Surdacka, Maria Slomka, Jacek Rolinski, Krzysztof Celinski, Agata Smolen, Mariusz Szczerbinski

**Affiliations:** ^1^Department of Gastroenterology with Endoscopy Unit, Medical University of Lublin, 8 Jaczewski Street, 20-954 Lublin, Poland; ^2^Department of Clinical Immunology, Medical University of Lublin, 4A Chodzki Street, 20-093 Lublin, Poland; ^3^Department of Mathematics and Biostatistics, Medical University of Lublin, 4 Jaczewski Street, 20-090 Lublin, Poland

## Abstract

*Background and aims*. There is growing evidence that white adipose tissue is an important contributor in the pathogenesis of alcoholic liver disease (ALD). We investigated serum concentrations of total adiponectin (Acrp30), leptin, and resistin in patients with chronic alcohol abuse and different grades of liver dysfunction, as well as ALD complications. *Materials and Methods*. One hundred forty-seven consecutive inpatients with ALD were prospectively recruited. The evaluation of plasma adipokine levels was performed using immunoenzymatic ELISA tests. Multivariable logistic regression was applied in order to select independent predictors of advanced liver dysfunction and the disease complications. *Results*. Acrp30 and resistin levels were significantly higher in patients with ALD than in controls. Lower leptin levels in females with ALD compared to controls, but no significant differences in leptin concentrations in males, were found. High serum Acrp30 level revealed an independent association with advanced liver dysfunction, as well as the development of ALD complications, that is, ascites and hepatic encephalopathy. *Conclusion*. Gender-related differences in serum leptin concentrations may influence the ALD course, different in females compared with males. Serum Acrp30 level may serve as a potential prognostic indicator for patients with ALD.

## 1. Introduction

White adipose tissue (WAT) represents an active endocrine organ that regulates body fat mass and energy balance. There is increasing evidence that WAT-derived adipokines may contribute to hepatic damage associated with fatty infiltration, inflammation, and fibrosis [1, 2]. Adiponectin, leptin, and resistin are the best described molecules in this class. Adiponectin is secreted exclusively from adipose tissue and circulates in different isoforms: trimers, of low molecular weight (LMW), hexamers (trimer dimer) of medium molecular weight (MMW), and multimeric high molecular weight (HMW) isoforms [3]. Leptin is expressed mainly by adipose tissue, although low levels have been detected in the placenta, skeletal muscle, gastric and mammary epithelium, and the brain [3]. Although resistin is secreted by human adipocytes, the most significant source appears to be blood mononuclear cells [3]. 

Plasma concentrations of adipokines have been investigated in patients with different liver disorders, that is, nonalcoholic fatty liver disease [4–6], type 1 autoimmune hepatitis [7], and viral hepatitis B [8] and C [9]. Alcoholic liver disease (ALD) is another one where adipokines may play pivotal role and represent a link between inflammation and metabolic state. However, the published data on the underlying pathophysiological mechanisms are ambiguous.

Therefore, the objective of the present study was to determine serum concentrations of total adiponectin (Acrp30), leptin, and resistin in patients with chronic alcohol abuse and different grades of liver dysfunction. We also investigated the adipokine correlation with traditional indicators of inflammation, liver laboratory parameters, and ALD complications. Furthermore, on the basis of above obtained results, we tried to select noninvasive predictors of the disease severity and ALD outcome. The prospective study was conducted in the Department of Gastroenterology of Medical University in Lublin, Poland. 

## 2. Material and Methods

One hundred forty-seven consecutive adult inpatients (pts) with ALD admitted to hospital due to the disease decompensation were prospectively recruited. The control group consisted of 30 healthy volunteers matched to the study group in terms of age and sex and recruited mostly among academics and trainees of Department of Gastroenterology, Medical University of Lublin. 

The diagnosis of ALD was based on clinical criteria such as a detailed patient history, typical symptoms and physical findings of chronic liver disease, laboratory values (elevated serum aminotransferases activity (normal range: ALT < 31 IU/L; AST < 34 IU/L), the AST/ALT ratio higher than 2), and imaging studies, in the setting of excessive alcohol intake (i.e., alcohol consumption exceeding 40 g/d for male and 20 g/d for female pts for a minimum of 6 months). In order to confirm alcohol misuse, the Alcohol Use Disorders Identification Test-Consumption (AUDIT-C) questionnaire was used [10]. An AUDIT score of ≥8 for men up to age 60, or ≥4 for women, or men over age 60 was considered a positive screening test. Positive AUDIT-C, in addition to the amount of alcohol consumption, was an inclusion criterion. Based on the AUDIT-C score, the alcohol consumption status was determined in both studied groups. Two individuals from the control group, who pledged total abstinence, were categorized as abstainers and eighteen who self-reported their consumption as no more than 20 g ethanol per day (10–16 drinks per month; 1 drink refers to 10 g of pure alcohol) as light drinkers. Quantity of drinking among risky, current drinkers from the ALD group ranged as follows: in females 40 g/d to more than 100 g/d and in males 50 g/d to more than 100 g/d. 

According to the study protocol, patients signed informed consent, completed an anamnesis (their medical history), and they answered the AUDIT-C questionnaire. 

Patients entering the study did not consume alcoholic beverages at least 24 hours prior to obtaining blood samples for laboratory tests. No one was treated with corticosteroids or pentoxifylline at the time of qualification. All data necessary for further analysis (i.e., the major demographic variables included age, gender, alcohol intake and the period of alcohol abuse, education, family history of alcoholism, employment status, treatment of ALD before admission, and comorbidities) were recorded, and the planned procedures including blood sampling were performed within 48 hours after hospital admission. Blood samples from all patients were collected at 07:30 am after a minimum 8-hour overnight fast in order to avoid the circadian and feeding impact on serum adipokine fluctuations. Basic laboratory tests included determination of

1. liver function parameters (alanine aminotransferase-ALT, aspartate aminotransferase-AST, alkaline phosphatase-AP, gamma-glutamyl transpeptidase-GGT, total bilirubin-Tbil, albumin, INR, and prothrombin time);
2. complete blood count (hemoglobin-Hgb, erythrocytes-RBC, platelets-PLT, and leukocytes-WBC);
3. parameters of renal function (creatinine-CREA, serum sodium level-Na);
4. the traditional markers of inflammation: neutrophils, neutrophil to lymphocytes count rate (NLR) and the level of C-reactive protein-CRP);
5. indicators of other etiology of chronic liver disease, that is, HBV and HCV infection (HBsAg, anti-HBc class IgM and IgG antibodies; anti-HCV antibodies), antinuclear, antismooth muscle, antimitochondrial antibodies, and markers of Wilson's disease and hemochromatosis as appropriate).

Based on the lab results, the baseline severity of liver dysfunction was determined according to the Child-Turcotte-Pugh (CTP) [11] and the Model of End-Stage Liver Disease (MELD) [12] criteria. For the score calculation, internet available calculators were used, that is, http://www.mayoclinic.org and http://potts-uk.com/livercalculator.html. 

Patients enrolled into the study were divided into subgroups according to:

1. gender;
2. age: ≥50 and < 50 years old;
3. the severity of liver dysfunction according to the CTP (classes A, B, C) and MELD (≥20 or < 20) scores;
4. the presence of ALD complications at the time of hospital admission, that is, ascites, hepatic encephalopathy (HE), oesophageal varices, cholestasis, and renal dysfunction.

Pts with any other severe diseases, that is, uncontrolled diabetes, heart failure, pulmonary insufficiency or malignancy at the time of inclusion were excluded. 

Symptoms of overt hepatic encephalopathy (HE) were classified according to West-Heaven criteria [13]. 

Cholestasis was defined based on the recommendations of the European Association for the Study of the Liver (EASL), that is, alkaline phosphatase (AP) greater than 1.5 times above the upper limit of normal (ULN) and the activity of *γ*-glutamyl transpeptidase (GGT), more than three times the ULN [14]. Ultrasonography of the abdomen was performed in order to confirm the presence of ascites and to rule out other causes of cholestasis (e.g., choledochal cyst and gallstones). Tests for antimitochondrial antibodies (AMAs) to exclude the diagnosis of primary biliary cirrhosis (PBC) were done, and drugs hepatotoxicity was ruled out.

Gastroscopy was performed in order to identify esophageal varices. Renal dysfunction was determined on the basis of elevated levels of serum creatinine (above the upper limit of normal, that is, 1.3 mg/dL). 

In some cases, a CT scan was also performed when any doubts about the nature of the pathology existed. 

Body mass index (BMI) and/or the waist-to-hip ratio (WHR) are commonly used for evaluation of body composition and fat mass. However, we did not apply those indicators due to their bias and inaccuracy of fat content assessment in ascitic cirrhotics. Ascites was present in 89 of 147 (60.5%) subjects included in the survey. Alcoholics with decompensated liver disease have both high prevalence of malnutrition and high BMI values due to fluid retention at the same time. Furthermore, some of our patients could not stand at the time of hospital admission, so the measure of their weight and height was impossible. The applicability of these constants for an estimation of body fat mass has often been questioned [15, 16]. 

The evaluation of plasma levels of selected adipokines (total adiponectin-Acrp30, leptin, and resistin) was performed with immunoenzymatic ELISA (enzyme linked immunosorbent assay) tests using commercially available kits (Quantikine ELISA kit, R & D Systems, USA). The study was conducted according to the procedure recommended by the producer and described in the attached materials. Measurements were made using VictorTM3 Reader (PerkinElmer, USA). 

The study protocol conforms to the ethical guidelines of the 1975 Declaration of Helsinki (6th revision, 2008) as reflected in a priori approval by the institutional review board of Medical University of Lublin. All subjects signed an informed consent form prior to the investigation.

## 3. Statistical Analysis

Statistical analysis was performed using the Statistica 10 software package (StatSoft, Poland). The distribution of the data in the groups was preliminarily evaluated by Kolmogorov and Smirnov test. The analysis showed a skewed distribution of all values; therefore, continuous variables were described as median with interquartile range, its 95% confidence interval (95% CI), and compared using Mann-Whitney *U*-test. Categorical variables are presented as numbers and percentage. Differences between categorical variables were assessed by Fisher's exact test or the *χ* ^2^ test with Yates correction for continuity, as appropriate. The differences of studied adipokine levels between CTP classes were analyzed using Kruskal-Wallis and multiple comparisons post hoc tests. Spearman's rank correlation test was used for the assessment of association between parameters of liver function, traditional indicators of inflammation, and studied biomarker serum levels. The receiver operating curves (ROCs) for significant adipokines, that is, Acrp30 and resistin were constructed to assess their areas under the curve (AUCs) and the best threshold values for predicting ALD complications. The method of DeLong et al. [17] for the calculation of the standard error of the AUC was used. The Youden index and its associated cut-off point were estimated for each adipokine [18]. AUCs of significant variables were compared to assess their accuracy in predicting the severity of liver dysfunction and the development of ALD complications. Multivariable logistic regression was applied in order to select independent predictors of advanced liver dysfunction and the occurrence of the disease complications. A two-sided *P* value of less than 0.05 was considered to be associated with statistical significance. 

## 4. **Results**

The survey population included 147 patients (107 males (72.8%) and 40 females (27.2%)). Their mean age was 49.56 ± 11.85 (range 26 to 74). The baseline characteristics of the study cohort are shown in Tables 1 and 2. 

Initially we performed an assessment of the adipokine serum levels in patients with ALD in comparison with healthy controls. The results indicated that serum Acrp30 and resistin levels were significantly higher in patients with ALD as compared to healthy controls. They are presented in Table 3. 

The next step was to compare the levels of studied biomarkers inside the ALD and control group according to gender. Serum Acrp30 (*P* = 0.004) and leptin (*P* = 0.03) concentrations were significantly higher in females compared to males from the control group. On the other hand, none of studied adipokines showed a significant difference according to gender in ALD group. Furthermore, significantly higher levels of Acrp30 in both sexes were found in ALD patients as compared to the control group. Serum leptin levels in females with ALD were significantly lower compared to controls. There were no significant differences in serum leptin levels in men in both groups. Resistin serum levels were significantly higher in both sexes in ALD patients as compared to the control group. The results are presented in Table 4.

Since aging alters body fat mass and its function, we evaluated serum adipokine levels in two age subgroups: ≥50 and < 50 years old. As expected, serum leptin concentration was significantly higher in the older patients in comparison with the younger subgroup. There were no age-related differences in serum levels of two other adipokines. The results are presented in Table 5.

It was found that only the level of Acrp30 among studied adipokines significantly increased with the severity of liver dysfunction classified according to both CTP and MELD scores. The results are presented in Tables 6 and 7.

The analysis of the association of adipokine serum levels and liver function parameters showed a significant, positive correlation between leptin and both aminotransferases and bilirubin serum levels in males. A positive correlation of leptin with albumin serum levels was observed in females. There was an inverse correlation of Acrp30 and resistin with serum albumin levels and a positive correlation of Acrp30 with AP and bilirubin levels in men. The results of analysis are shown in Table 8.

We found a significant positive correlation of serum resistin level and both the white blood cells count and CRP level. Serum leptin concentrations showed a weak inverse correlation with the white blood cells count. The results of the above analysis are presented in Table 9.

The next step of the study was carried out to compare the level of studied adipokines in ALD patients divided according to the presence of the disease complications. For adipokines which serum concentrations differed significantly in subgroups of patients selected according to the severity of liver dysfunction (MELD ≥ 20) and the complications of ALD, the areas under the curve (AUCs) were checked, and the diagnostic accuracy of studied variables for the association with complications of the disease was compared. Serum Acrp30 levels were identified as an independent predictor of advanced liver dysfunction (MELD ≥ 20) and the development of ascites and HE. Serum resistin levels lost their significance for renal dysfunction when adjusted for other variables. None of the studied adipokines was independently associated with cholestasis and esophageal varices. Above results are summarized in Tables 10, 11, and 12.

## 5. **Discussion**

Excessive and chronic alcohol consumption leads to inflammation in adipose tissue, insulin resistance, and hepatic steatosis [19]. Therefore, the exploration of the pathogenic mechanisms of ALD should include the role of adipose tissue secretion and adipokines. In addition, alcohol abuse is associated with impaired energy intake and expenditure, as well as increased catabolism. All above mentioned processes are modulated by adipokines. The recent study of Zhong et al. [20] demonstrated a significant loss of white adipose tissue (WAT) in a mouse model of alcoholic steatosis. It suggests that WAT dysfunction can directly impact hepatic lipid homeostasis by reverse triglyceride transport. Clinical studies have shown that lower fat mass is associated with higher liver fat content in alcoholics [21]. Those reports prompted us to design a study in order to explain the association of serum adipokine concentrations with the severity of liver dysfunction and ALD complications. Results obtained in the present study confirmed the crucial role of WAT endocrine secretion in the pathogenesis of ALD. Significantly higher levels of two adipokines, Acrp30 and resistin were found in patients with ALD compared to the control subjects. Unexpectedly, there were no differences in leptin levels in both studied groups (Table 3). 

The analysis of data by gender showed significantly higher levels of Acrp30 and leptin in females from the control group in comparison with the level of both adipokines in males (Table 4). Our results are consistent with other reports regarding gender-related differences of serum adipokine concentrations [22]. However, no significant difference in the level of three studied adipokines in relation to sex in the ALD group was observed. In contrast, a significantly lower leptin level was found in females with ALD in comparison with healthy controls. The result corresponds to the above mentioned Zhong et al. report [20] indicating possible reduction of the body fat content. When comparing the severity of liver dysfunction in both sexes, females showed significantly higher scores of the CTP and MELD (Table 2). Hypoleptinemic states are associated with increased risk of infection [23, 24]. Therefore it may alter the course of ALD in both sexes.

We are tempted to speculate that metabolic alterations caused by ethanol in the course of ALD, by modulating secretion of leptin, might be responsible for different clinical presentation of the disease in females and males. It has been already reported by Röjdmark et al. [25] that ingestion of moderate amounts of alcohol had an inhibitory effect on leptin secretion in normal subjects. The effect might be direct rather than indirect, since several factors known to affect leptin were not influenced by alcohol in their study. Furthermore, leptin levels increase during abstinence, and this may be related to a reduction of dopaminergic action in mesolimbic system [26]. Gender-related differences observed in our study are also consistent with the results obtained by Dammann et al. [27] who observed no significant effect of acute moderate alcohol intake on leptin levels in healthy male volunteers. 

Previous reports concerning leptin levels in human alcoholics are inconclusive. The data were highly divergent and dependent on the population studied. Some of them pointed out increased [28, 29], and others lowered leptin concentrations in the peripheral blood [30].

Greco et al. [31] also observed higher serum leptin levels in healthy females compared to healthy males in the control group. However, unlike in the present work, they reported elevated levels of leptin in women with alcoholic cirrhosis child class C. The above mentioned study revealed a significant reduction in leptin serum levels in posthepatitis cirrhotic patients. The discrepancy between our results could be likely explained by evaluation of patients in different stages of ALD. The level of leptin in our cohort was assessed during an acute phase of liver disease with coexistence of signs of hypermetabolism and systemic inflammatory activation. In contrast to present report, the above-cited study was performed in patients with a stable chronic phase of the disorder.

De Timary et al. [32] proposed a dual model for regulation of energy intake in alcohol-dependent subjects. They showed that alcohol accelerates metabolism and decreases fat mass and leptin levels in individuals consuming above 12.5 kcal/kg/day of alcohol (lower leptin level was observed in females with ALD in our study). For individuals consuming below 12.5 kcal/kg/day of alcohol, alcohol intake is compensated for by a decrease in nonalcoholic nutrient intakes, probably due to changes in metabolic and satiety factors. 

On the other hand, Campillo et al. [28] observed an increase in leptin levels in the blood after nutritional therapy for patients with alcoholic cirrhosis in parallel to the liver function improvement.

Similar profile of serum adipokine concentrations that is elevated Acrp30 and resistin, and decreased leptin was reported in patients with inflammatory bowel disease [33].

We investigated the levels of adipokines in subgroups of ALD patients with varying severity of hepatic dysfunction defined according to the criteria of the CTP and MELD. We found that serum Acrp30 levels significantly rose with the degree of liver function impairment (Tables 6 and 7). Multivariable analysis confirmed its independent impact on the severity of liver dysfunction (MELD ≥ 20) (Table 12). It is in contrast to nonalcoholic fatty liver disease (NAFLD), where serum adiponectin concentrations were reported to be low and negatively related to necroinflammatory injury [34, 35]. On the other hand, a great body of evidence suggests a beneficial and protective effect of Acrp30 in liver injury in mice [36, 37]. Its administration reduced ROS production stimulated by LPS and expression of Tumor Necrosis Factor *α* (TNF *α*) in Kupffer cells [38]. The mechanism by which adiponectin levels and action differ between humans and rodents is still unknown. 

Correlations were found in men between serum Acrp30 levels and the parameters of liver function: positive with the level of bilirubin, inverse with albumin level (Table 8). These results are consistent with previous indices of positive Acrp30 association with the severity of liver dysfunction (CTP and MELD). In addition, a weak inverse correlation between serum resistin concentrations and the level of albumin in ALD group was observed.

Furthermore, the results of our study indicate the presence of adipokine association with major complications of the disease (Table 10). Again Acrp30 seems to play a pivotal role. Its serum level was significantly elevated in patients with ascites and encephalopathy. Multivariable analysis confirmed independent impact of Acrp30 on both complications (Table 12). 

The borderline statistical significance (*P* = 0,054) of leptin level differences in subgroups with and without ascites was found and might suggest its negative influence on the evolution of ALD (Table 10). Nevertheless, the multivariable analysis failed to prove its impact on the development of the above complication. As observed in our study, also Brennan et al. [39] did not find any significant association of leptin concentration with morbidity and mortality for cardiovascular disease in women with diabetes. Conversely, reports of other authors indicate that leptin deficiency may enhance the sensitivity to the toxic effects of inflammatory factors, including endotoxin and TNF *α* [40–42].

Serum resistin levels were significantly increased in the subgroups with symptoms of cholestasis and renal dysfunction (Table 9). Nevertheless, they lost their significance when adjusted for other studied variables. The presence of elevated levels of resistin was previously reported in the course of chronic kidney disease [43–45]. On the other hand, Menzaghi et al. [46] demonstrated that resistin may play an important role in modulating the kidney function in healthy subjects. In the present study, the multivariable analysis failed to confirm its independent effect on both the above-mentioned complications. 

Suggestions about the prognostic value of adiponectin and resistin in the assessment of severity and outcome of inflammatory diseases and cancer appeared in several recent publications [47–51]. We measured the plasma levels of total adiponectin (Acrp30), which has been reported to be more useful than HMW for assessing mortality risk. The high plasma concentration of Acrp30 was an independent prognostic predictor in chronic heart failure patients with normal BMI [52]. Higher concentrations of total adiponectin were also associated with heart failure and mortality among patients with existing ischemic heart disease [53].

The development of ALD is believed to combine metabolic and inflammatory activity, so the assessment of the relationship between serum levels of selected adipokines and inflammatory markers was of vast interest. We speculate that significantly increased serum levels of Acrp30 and resistin in the studied cohort may be an indicator of systemic inflammatory activation in the course of the disease. The data from the literature suggest that the levels of both adipokines rise in chronic inflammation [54–57]. As described elsewhere, leptin also affects immune and inflammatory functions. We found its weak inverse correlation with the white blood cells count. 

On the other hand, an increase in Acrp30 concentration in the blood of patients with ALD may reflect the defense mechanism rather than inflammatory response due to its known anti-inflammatory properties [58]. Furthermore, the level of Acrp30 in the studied group did not show any correlation with the traditional parameters of inflammation (Table 9). The results correspond to the general belief about its anti-inflammatory potential. It was reported that Acrp30 deficiency leads to persistent subclinical inflammation in the course of obesity, nonalcoholic fatty liver disease (NAFLD), ischemic heart disease, and type 2 diabetes [59–63]. 

In contrast, increased levels of serum adiponectin have been observed in inflammatory and/or autoimmune disorders which show no association with obesity and positive energy balance. Those include rheumatoid arthritis, lupus erythematosus, and inflammatory bowel diseases [64, 65]. It appears that secretion of Acrp30 may be differently regulated depending on the pathogenesis of underlying disease, in particular on its association with energy balance (overweight or obesity). 

Furthermore, Behre [66] hypothesized that in subjects with an energy deficit in the course of anorexia, cachexia, type 1 diabetes, and renal failure, when the blood level of adiponectin increased, it might have a protective role and adapt the body during fasting. The results obtained in the present study are consistent with such an interpretation of the facts. The patients with ALD, regardless of their gender, had significantly elevated plasma levels of Acrp30 compared with the control group (Table 4). It may be explained by malnutrition frequently occurring in the course of the disease. 

Proinflammatory properties of resistin in our study were confirmed by a positive correlation of its serum concentration with the white blood cells count and CRP level (Table 9). Yoshino et al. [57] reported that the plasma levels of resistin and leptin positively correlated with the CRP level in patients with rheumatoid arthritis. Similar observations in patients with Kawasaki disease come from Kemmotsu et al. report [67]. The level of CRP was significantly associated with an increased level of resistin in that study. The results of Trzeciak-Ryczek et al. [68] from *in vitro* studies revealed that macrophage stimulation with lipopolysaccharide or pro-inflammatory cytokines (IL-1, IL-6 and TNF) considerablly increased resistin production during infection. The presented data support the hypothesis that resistin may become a new marker of inflammation.

Our study has some limitations. It was a single-center trial, and it should be emphasized that due to the insufficient sample size, our results before their generalization need to be confirmed in prospective, multicenter studies. Such validation may help to eliminate possible errors resulting from research techniques and subjective differences in the selection of the study population. Another limitation of the study was lack of estimation of the body fat content by objective and validated measures. The newest diagnostic option is dual-energy X-ray absorptiometry (DEXA), which may be used as a gold standard with high accuracy [69]. Nevertheless, the procedure was unavailable during our study. Furthermore, the control group of healthy abstainers and light drinkers (≤20 g/day) was used as the reference category for alcohol consumption in the study. More often disease-free drinkers are included as a comparison cohort to control for the possible effect of alcohol consumption. Alcohol intake was self-reported in our study. Participants had to determine the amount of alcohol consumed in drinks per day, week, and/or month. Since these values (i.e., the volume and alcohol concentration of a beverage) are not easy to estimate, the real ethanol intake may lack precision [70]. A study by Stockwell and Stirling [71] also showed that most people are not able to accurately assess the volume and power of a drink. In countries like Poland, alcohol intake patterns vary considerably by regions and beverage type, so researchers should pay special attention to careful assessment of drink types and sizes for accurate alcohol consumption estimation. 

In conclusion, the results obtained in the present study confirmed the active participation of WAT in the pathogenesis of ALD. We speculate that gender-related differences in serum leptin concentrations may influence the ALD course, different in females compared to males. It seems that, beyond their metabolic influence, adipokines may play a role of inflammatory modulators. An independent association of the high serum Acrp30 level with advanced liver dysfunction, as well as the development of ALD complications (i.e., ascites and hepatic encephalopathy) may indicate its potential role as an ALD prognostic indicator. In addition, due to the inverse serum concentration, Acrp30 may become a relevant marker for differentiation between ALD and NAFLD. The association of resistin levels with renal dysfunction in ALD patients should be further elucidated. 

Good availability of serum biomarkers makes them a promising, minimally invasive diagnostic tool for the possible widespread use in the clinical practice. It appears that the determination of molecules involved in different stages of the disease evolution may help to generate a reliable diagnostic algorithm in the future. Whether combination therapy with adipokines may have a role in ALD patients is being greatly anticipated. The modulation of their secretion might be considered as a tempting therapeutic procedure but needs to be investigated in detail.

## Figures and Tables

**Table 1:** Baseline characteristics of the study population*.

	ALD group *n* = 147	Control group *n* = 30	*P*
Age (years)			
Males	49.84 ± 11.53	44.31 ± 10.23	0.09
Females	48.82 ± 9.94	43.11 ± 8.43	0.10

Gender			
Males	107 (72.8%)	17 (56.7%)	0.12
Females	40 (27.2%)	13 (43.3%)	

*ALD: alcoholic liver disease.

**Table 2:** Characteristics of patients with ALD based on their gender*.

	ALD group (*n* = 147)						*P*
	Females (*n* = 40)			Males (*n* = 107)		
	Median	95% CI	25–75 P	Median	95% CI	25–75 P
Age years	51.00	48.03–54.96	45.00–56.00	51.00	48.00–52.49	40.00–60.00	0.19
ALT IU/L	39.50	28.03–44.93	23.00–47.00	56.00	50.00–69.00	35.25–84.00	**0.004**
ASP IU/L	100.50	78.45–114.90	66.00–120.00	110.00	78.51–131.00	64.50–189.00	0.72
AP IU/L	118.50	111.68–156.27	105.00–179.00	129.00	118.00–148.00	79.00–223.00	0.62
GGT IU/L	415.00	174.00–543.00	172.00–772.00	359.00	200.50–504.88	93.00–1066.00	**0.020**
T-Bil mg/dL	4.20	3.51–5.27	3.30–8.40	3.00	1.75–4.00	1.10–8.10	0.70
Alb g/dL	3.10	2.70–3.29	2.63–3.50	3.20	3.00–3.30	2.73–3.61	0.12
INR	1.45	1.39–1.64	1.31–1.71	1.21	1.16–1.30	1.07–1.43	**0.034**
Crea mg/dL	0.80	0.70–0.80	0.70–1.00	0.90	0.90–1.00	0.80–1.10	0.51
Na mEq/L	139.00	136.03–140.96	134.00–141.00	138.00	136.51–139.00	134.00–140.00	0.38
Hgb g/dL	11.20	10.34–11.50	9.70–12.00	12.10	11.60–12.70	10.30–13.50	**<0.001**
RBC × 10^6^ kom/uL	3.17	3.08–3.50	2.86–3.52	3.86	3.57–3.97	3.15–4.11	**<0.001**
PLT × 10^3^ kom/uL	135.50	114.38–137.96	97.00–251.00	136.00	116.00–166.46	80.00–202.00	0.81
WBC × 10^3^ kom/uL	8.12	5.42–11.63	4.89–13.04	7.12	6.30–8.28	5.01–10.80	0.75
NEUT × 10^3^ kom/uL	8.44	3.20–8.97	2.57–13.51	5.02	4.19–6.10	2.91–7.92	**0.053**
NLR	4.38	2.34–4.52	2.34–7.63	3.47	3.26–4.45	2.13–6.04	0.12
CRP mg/L	17.33	16.19–33.14	5.98–42.17	17.53	13.40–21.30	5.01–43.00	0.58
mDF	17.35	12.00–22.96	9.00–28.00	9.00	6.00–12.00	4.00–16.74	0.21
MELD	17.50	15.03–18.00	12.00–20.00	15.00	14.00–16.00	11.00–17.00	**0.047**
CTP	9.50	9.00-10.00	8.00–10.00	7.00	7.00–8.00	7.00–9.00	**<0.001**

*Alb: albumin (normal range (NR) 3.2–4.8); ALT: alanine aminotransferase (NR) < 31); AP: alkaline phosphatase (NR 45–129); AST: aspartate aminotransferase (NR < 34); Crea: creatinine (NR 0.5–1.1); CRP: C-reactive protein (NR 0.0–5.0); CTP-Child-Turcotte-Pugh score; GGT: gamma-glutamyl transpeptidase (NR < 50.0); Hgb: hemoglobin (NR 14.0–18.0); INR: International Normalized Ratio (NR 0.8–1.2); MELD: Model for End-Stage Liver Disease; Na: sodium (NR 136–145); NEUT: neutrophils (NR 1.8–7.7); NLR: neutrophil to lymphocyte ratio; PLT: platelets (NR 130–400); 25–75 P: percentiles, RBC: red blood cells (NR 4.5–6.1); T-Bil: total bilirubin (NR 0.3–1.2); WBC: white blood cells (NR 4.8–10.8).

**Table 3:** Comparison of serum adipokine levels in ALD patients and the control group*.

	Adipokines						*P*
	ALD group (*n* = 147)			Control group (*n* = 30)		
	Median	95% CI	25–75 P	Median	95% CI	25–75 P
Acrp30 *µ*g/mL	18.69	16.86–24.03	13.17–44.94	6.38	4.83–8.19	4.06–8.68	**<0.0001**
Leptin ng/mL	8.80	6.80–10.41	2.75–13.96	8.76	5.32–16.94	5.13–17.31	0.36
Resistin ng/mL	16.91	15.27–18.84	10.63–29.01	8.87	8.36–10.71	8.36–11.41	**0.0001**

*Acrp30: total adiponectin; CI: confidence interval, 25–75 P: percentiles.

**Table 4:** Comparison of serum adipokine levels in females and males with ALD and the control group.

	Females						*P*
	ALD group (*n* = 40)			Control group (*n* = 13)		
	Median	95% CI	25–75 P	Median	95% CI	25–75 P
Acrp30 *µ*g/mL	17.93	15.76–30.09	13.06–48.37	7.78	6.35–24.81	6.71–24.81	**0.02**
Leptin ng/mL	6.80	3.30–11.00	2.55–13.00	16.64	5.13–42.10	5.13–42.10	**0.009**
Resistin ng/mL	16.18	11.42–17.05	8.89–19.22	9.88	6.30–10.73	6.30–10.45	**0.01**

	Males						*P*
	ALD group (*n* = 107)			Control group (*n* = 17)		
	Median	95% CI	25–75 P	Median	95% CI	25–75 P

Acrp30 *µ*g/mL	18.89	16.93–24.83	13.17–43.94	4.06	3.59–6.38	3.59–6.38	**<0.0001**
Leptin ng/mL	9.45	7.82–10.88	3.40–14.96	8.57	1.79–9.17	1.79–8.97	0.17
Resistin ng/mL	17.69	15.36–21.58	11.08–31.17	8.87	8.36–15.12	8.36–15.12	**0.005**

**Table 5:** Comparison of serum adipokine levels according to the age of patients with ALD.

	Adipokines in ALD group						*P*
	Age ≥ 50 (*n* = 83)			Age < 50 (*n* = 64)		
	Median	95% CI	25–75 P	Median	95% CI	25–75 P
Acrp30 *µ*g/mL	23.11	16.20–40.14	11.49–70.52	18.07	16.60–23.42	14.13–32.25	0.32
Leptin ng/mL	10.69	8.51–12.33	3.03–15.57	6.57	4.77–8.81	2.67–10.88	**0.02**
Resistin ng/mL	17.03	16.36–19.04	13.48–29.69	13.82	11.05–21.20	8.50–28.62	0.13

**Table 6:** Serum adipokine levels in patients with ALD according to the CTP class.

	CTP class									*P*
	Class A (*n* = 30)			Class B (*n* = 73)			Class C (*n* = 44)		
	Median	95% CI	25–75 P	Median	95% CI	25–75 P	Median	95% CI	25–75 P
Acrp30 *µ*g/mL	15.76	8.97–22.50	7.80–29.60	23.33	17.06–29.15	14.67–45.78	23.11	16.73–95.85	14.92–156.63	**0.02**
Leptin ng/mL	8.45	3.03–10.39	2.00–11.88	9.88	7.84–10.94	5.25–14.01	5.28	3.04–12.95	2.13–16.15	0.34
Resistin ng/mL	14.40	9.39–27.59	8.47–32.33	16.99	14.80–25.32	10.84–31.44	16.77	14.57–18.89	13.33–21.26	0.59

**Table 7:** Serum adipokine levels in patients with ALD according to the MELD score.

	MELD						*P*
	<20 points (*n* = 117)			≥20 points (*n* = 30)		
	Median	95% CI	25–5 P	Median	95% CI	25–75 P
Acrp30 *µ*g/mL	17.93	15.97–23.33	12.49–42.04	37.60	19.29–157.63	17.06–161.19	**0.01**
Leptin ng/mL	8.95	6.80–10.88	2.69–14.52	7.56	3.21–10.39	2.91–12.41	0.51
Resistin ng/mL	16.64	14.40–18.17	10.23–28.08	19.36	14.57–27.92	13.37–63.53	0.08

**Table 8:** Analysis of the correlation between serum adipokine levels and liver function parameters.

	Females (*n* = 40)		Males (*n* = 107)		Resistin
	Acrp30	Leptin	Acrp30	Leptin	
ALT					
*Rho	−0.08	0.30	−0.14	0.25	−0.15
*P*	0.62	**0.06**	0.14	**0.01**	0.06
ASP					
Rho	−0.14	0.12	−0.13	0.25	−0.11
*P*	0.40	0.46	0.18	**0.01**	0.20
AP					
Rho	−0.07	−0.14	0.21	−0.15	−0.01
*P*	0.67	0.42	**0.04**	0.15	0.92
GTP					
Rho	0.03	0.02	0.08	0.16	−0.13
*P*	0.87	0.92	0.44	0.34	0.14
Albumin					
Rho	−0.20	0.38	−0.27	0.13	−0.17
*P*	0.22	**0.02**	**0.007**	0.18	**0.04**
T-Bilirubin					
Rho	0.14	0.05	0.23	0.27	−0.03
*P*	0.37	0.76	**0.02**	**0.006**	0.71
INR					
Rho	0.23	−0.28	0.14	0.01	0.09
*P*	0.15	0.07	0.15	0.95	0.29

*Rho: Spearman's rank correlation coefficient.

**Table 9:** Analysis of a correlation between serum adipokine levels and traditional markers of inflammation.

	WBC	Neutrophils	NLR	CRP
Acrp30				
*Rho	−0.05	−0.07	−0.13	−0.00
*P*	0.52	0.40	0.11	0.98
Leptin				
Rho	−0.17	−0.13	−0.07	0.00
*P*	**0.04**	0.13	0.41	0.96
Resistin				
Rho	0.23	0.14	0.11	0.31
*P*	**0.004**	0.09	0.17	**0.0002**

*Rho: Spearman's rank correlation coefficient.

**Table 10:** Serum adipokine levels in patients with ALD according to the presence of the disease complications.

	Ascites						*P*
	Absent (*n* = 58)			Present (*n* = 89)		
	Median	95% CI	25–75 P	Median	95% CI	25–75 P
Acrp30 *µ*g/mL	16.94	14.78–23.04	10.16–29.62	23.33	17.93–37.81	14.55–74.20	**0.013**
Leptin ng/mL	10.47	8.42–11.89	6.34–15.13	6.80	4.40–9.88	2.20–13.32	**0.054**
Resistin ng/mL	17.15	11.62–24.88	9.33–32.27	16.75	15.13–18.54	11.36–28.08	0.70

	Hepatic encephalopathy						*P*
	Absent (*n* = 127)			Present (*n* = 20)		
	Median	95% CI	25–75 P	Median	95% CI	25–75 P

Acrp30 *µ*g/mL	17.93	16.08–23.38	11.76–41.82	55.65	23.11–164.21	22.62–164.55	**0.003**
Leptin ng/mL	8.82	6.80–10.49	2.75–14.27	6.42	3.06–12.63	2.76–13.33	0.54
Resistin ng/mL	16.37	14.39–18.91	10.30–29.69	18.17	16.03–20.86	15.83–21.36	0.55

	Oesophageal varices						*P*
	Absent (*n* = 60)			Present (*n* = 87)		
	Median	95% CI	25–75 P	Median	95% CI	25–75 P

Acrp30 *µ*g/mL	17.55	15.20–23.11	11.76–31.42	23.61	17.91–33.14	14.68–51.66	0.16
Leptin ng/mL	8.88	3.66–11.88	2.00–13.94	8.38	5.97–10.13	3.23–13.56	0.69
Resistin ng/mL	18.73	14.40–22.34	9.09–27.24	15.83	13.46–17.53	11.14–28.31	0.59

	Cholestasis						*P*
	Absent (*n* = 117)			Present (*n* = 30)		
	Median	95% CI	25–75 P	Median	95% CI	25–75 P

Acrp30 *µ*g/mL	18.22	16.33–29.60	10.60–68.81	20.10	14.13–25.37	11.76–41.45	0.77
Leptin ng/mL	8.94	7.03–10.69	3.84–15.14	5.23	2.27–10.92	2.00–11.90	0.07
Resistin ng/mL	15.29	13.37–16.99	9.28–26.81	19.74	17.11–31.21	13.46–33.87	**0.02**

	Renal dysfunction						*P*
	Creatinine < 1.3 mg/dL (*n* = 125)			Creatinine ≥ 1.3 mg/dL (*n* = 22)		
	Median	95% CI	25–75 P	Median	95% CI	25–75 P

Acrp30 *µ*g/mL	18.22	16.65–24.07	11.50–44.27	22.63	17.03–46.19	16.33–161.19	0.12
Leptin ng/mL	9.23	6.80–10.91	3.11–15.00	7.56	2.00–9.08	2.00–10.09	0.18
Resistin ng/mL	16.18	13.46–17.87	10.23–26.08	29.22	16.98–39.77	16.75–63.53	**0.001**

**Table 11:** Comparison of the diagnostic accuracy (AUCs) of single variables in the diagnosis of advanced liver dysfunction (MELD ≥ 20) and ALD complications. (univariable analysis)*.

Complication of ALD	Variable	*P* value	AUC (95% CI)	SE
MELD ≥ 20	Acrp30	0.002	0.652 (0.569–0.728)	0.060
	CRP	0.004	0.609 (0.527–0.686)	0.058
	RBC	0.003	0.675 (0.596–0.747)	0.055
	WBC	0.0003	0.656 (0.577–0.730)	0.061
	Ascites	0.003	0.652 (0.572–0.725)	0.050
	HE	<0.0001	0.666 (0.587–0.739)	0.058

Ascites	Acrp30	0.015	0.621 (0.537–0.700)	0.046
	Albumin	<0.0001	0.819 (0.748–0.877)	0.036
	ALT	0.0001	0.710 (0.633–0.779)	0.041
	AST	0.003	0.606 (0.526–0.683)	0.047
	INR	<0.0001	0.808 (0.739–0.866)	0.036
	RBC	0.002	0.663 (0.584–0.736)	0.044
	WBC	0.008	0.597 (0.517–0.674)	0.045

HE	Acrp30	0.002	0.704 (0.623–0.776)	0.071
	AP	0.006	0.652 (0.567–0.730)	0.071
	albumin	0.005	0.686 (0.605–0.759)	0.055
	T-bilirubin	0.0001	0.770 (0.697–0.833)	0.048
	INR	0.0001	0.737 (0.661–0.804)	0.059
	PLT	0.035	0.633 (0.553–0.708)	0.057
	Ascites	0.012	0.646 (0.567–0.720)	0.056

Renal dysfunction	Resistin	0.001	0.721 (0.641–0.792)	0.060
	Albumin	0.034	0.654 (0.572–0.729)	0.059
	AST	0.042	0.601 (0.521–0.678)	0.062
	AP	0.030	0.677 (0.593–0.753)	0.068
	Na	0.012	0.588 (0.508–0.666)	0.080
	CRP	0.001	0.714 (0.636–0.784)	0.060
	WBC	0.011	0.689 (0.611–0.760)	0.053
	RBC	0.031	0.688 (0.610–0.759)	0.059

*AUC: area under the ROC curve; SE: standard error; CI: confidence interval.

**Table 12:** Independent predictors of advanced liver dysfunction (MELD ≥ 20) and ALD complications (multivariable analysis)*.

Complication of ALD	Variable	*P* value	Adjusted OR (95% CI)	AUC (95% CI)	SE
MELD ≥ 20	Acrp30	0.001	1.013 (1.005–1.022)	0.873 (0.807–0.923)	0.041
	CRP	0.004	1.017 (1.005–1.029)		
	WBC	0.030	1.119 (1.011–1.238)		
	HE	0.0009	8.184 (2.373–28.224)		

Ascites	Acrp30	0.050	1.009 (1.000–1.019)	0.902 (0.840–0.946)	0.028
	albumin	0.0001	0.095 (0.030–0.300)		
	ALT	0.003	0.978 (0.964–0.993)		
	INR	0.003	22.137 (2.780–176.287)		
	RBC	0.021	2.962 (1.177–7.452)		

HE	Acrp30	0.010	1.011 (1.003–1.019)	0.830 (0.757–0.889)	0.048
	albumin	0.050	0.323 (0.105–0.998)		
	T-bilirubin	0.001	1.116 (1.045–1.193)		

*AUC: area under the ROC curve, CI: confidence interval, OR: Odds ratio, SE: standard error, HE: hepatic encephalopathy.
